# Observation of possible topological in-gap surface states in the Kondo insulator SmB_6_ by photoemission

**DOI:** 10.1038/ncomms4010

**Published:** 2013-12-18

**Authors:** J. Jiang, S. Li, T. Zhang, Z. Sun, F. Chen, Z.R. Ye, M. Xu, Q.Q. Ge, S.Y. Tan, X.H. Niu, M. Xia, B.P. Xie, Y.F. Li, X.H. Chen, H.H. Wen, D.L. Feng

**Affiliations:** 1 State Key Laboratory of Surface Physics, Department of Physics, and Advanced Materials Laboratory, Fudan University, Shanghai 200433, China; 2 National Laboratory of Solid State Microstructures and Department of Physics, Nanjing University, Nanjing 210093, China; 3 Department of Physics, University of Science and Technology of China, Hefei 230026, China; 4 National Synchrotron Radiation Laboratory, University of Science and Technology of China, Hefei 230029, China; 5 Hefei National Laboratory for Physical Science at Microscale, University of Science and Technology of China, Hefei 230026, China; 6 These authors contributed equally to this work

## Abstract

SmB_6_, a well-known Kondo insulator, exhibits a transport anomaly at low temperature. This anomaly is usually attributed to states within the hybridization gap. Recent theoretical work and transport measurements suggest that these in-gap states could be ascribed to topological surface states, which would make SmB_6_ the first realization of topological Kondo insulator. Here by performing angle-resolved photoemission spectroscopy experiments, we directly observe several dispersive states within the hybridization gap of SmB_6_. These states show negligible *k*_*z*_ dependence, which indicates their surface origin. Furthermore, we perform photoemission circular dichroism experiments, which suggest that the in-gap states possess chirality of the orbital angular momentum. These states vanish simultaneously with the hybridization gap at around 150 K. Together, these observations suggest the possible topological origin of the in-gap states.

## Abstract (web summary)

The Kondo insulator samarium hexaboride exhibits low-temperature transport anomalies, which might be due to topological surface states. Here Jiang *et al.* perform angle-resolved photoemission and its circular dichroism measurements, which suggest that the anomalies might be of topological origin.

## Graphical abstract

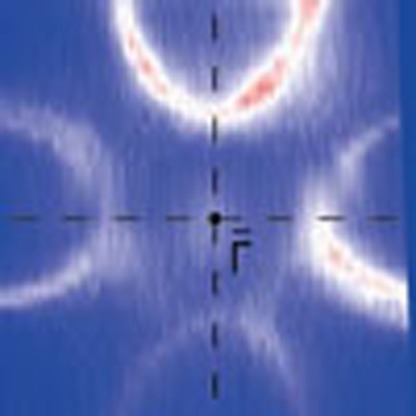

Kondo insulators (KIs) or heavy-fermion semiconductors^1^,^2^,^3^ are exotic materials with strong electron correlations, in which the localized *4f* electrons give rise to novel ground states. SmB_6_ is a well-known KI. At high temperature, the many-body interactions between the local spins of Sm ions and conduction electrons (the Kondo screening) are weak, and the system is a correlated metal. With decreasing temperature, an energy gap develops due to the hybridization of *4f* bands and conduction *5d* bands^1^,^2^,^3^, featured by a rapidly rising resistance. However, there is a long-standing puzzle: its resistivity does not diverge but saturates at temperatures below 4 K (refs 4, 5, 6). The residual conductivity was attributed to some in-gap states, evidenced by optical, neutron scattering, specific heat and angle-resolved photoemission spectroscopy (ARPES) experiments^7^,^8^,^9^,^10^. Although various models have been proposed to address the in-gap states^5^,^11^, their exact nature remains illusive after decades of studies.

Recently, the novel concept of topological KI (TKI) may shed new light on the in-gap states in SmB_6_ (refs 12, 13). In the so-called topological insulator (TI) such as Bi_2_Se_3_ or Bi_2_Te_3_ (refs 14, 15), there will be surface states that are protected by band topology from impurities that do not break time-reversal symmetry. Although the usual TIs are defined in weakly interacting systems, KIs with strong correlations can also be topologically classified. A topologically non-trivial KI could be realized by strong spin-orbit coupling associated with a hybridization band gap^12^,^13^. If SmB_6_ is a TKI as predicted^12^,^13^,^16^,^17^, there will be surface states that naturally explains the origin of in-gap states and the residual conductivity. In particular, unlike traditional TI materials in which surface transport was usually concealed by the defect-induced bulk carriers, SmB_6_ seems to possess a true insulating bulk that will make it a promising candidate material for future spintronics applications^18^. Several recent experiments have observed pronounced surface-dominating transport in SmB_6_ (refs 10, 18, 19, 20, 21) suggesting the TKI scenario. However, direct electronic structure evidences for these potential topological surface states are highly demanded for the understanding of their exact nature.

In this letter we present an ARPES study of SmB_6_ single crystals. We observe several bands within the hybridization gap of SmB_6_, which show negligible *k*_*z*_ dependence, indicative of their surface origin. Furthermore, the photoemission circular dichroism (CD) of these in-gap states suggests the chirality of the orbital angular momentum (OAM), and these states vanish simultaneously with the hybridization gap around 150 K. Our results thus strongly suggest their possible topological origin.

## Results

### Sample characterization and valence band structure

**Figure 1:**
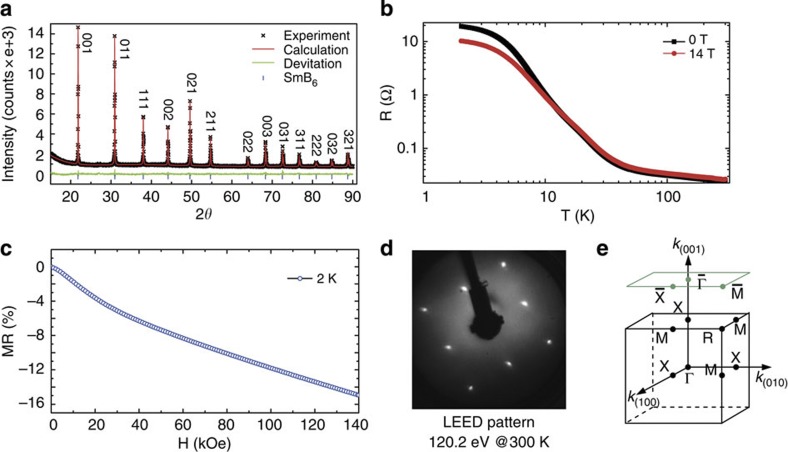
SmB_6_ sample characterization. (**a**) Powder X-ray diffraction pattern of an SmB_6_ crystal. Solid line is the Rietveld fitting using the TOPAS software. The s.d. is negligible. (**b**) The temperature dependence of resistance at zero field and 14 T. A negative magnetoresistance is observed in the low temperature region, while it becomes positive in the high temperature region. (**c**) The magnetoresistance of SmB_6_ taken at 2 K. A negative magnetoresistance is obvious here. (**d**) LEED pattern of the cleaved SmB_6_ (001) surface. Bright spots in square lattice reflect the pristine 1 × 1 surface, with a lattice constant of 4.13 Å. (**e**) Bulk and surface projected Brillouin zone of SmB_6_ and the high symmetry points.

Sample preparation and experimental details are described in the Methods section. Figure 1a shows the powder X-ray diffraction pattern of SmB_6_ taken after grinding the single crystals into powder. No evident secondary phase is observed, showing the high quality of the crystals. The resistivity of our SmB_6_ sample exhibits a sharp increase below 50 K and starts to saturate below 5 K (Fig. 1b), similar to previous reports^4^,^5^,^6^. As shown in Fig. 1b,c, magnetoresistance is clearly present, which is negative below ~20 K and positive at higher temperatures. The negative magnetoresistance can be understood in the context of Kondo effect, where the Kondo singlet will be partially broken by the magnetic field, which releases the localized *d*-band electrons and lowers the resistivity. In contrast, the positive magnetoresistance at high temperatures might be induced by the orbital scattering effect. Figure 1d shows the low-energy electron diffraction (LEED) pattern of the cleaved surface. Clean 1 × 1 pattern of the (001) surface is observed. The bulk and projected Brillouin zones of the (001) surface are shown in Fig. 1e.

**Figure 2:**
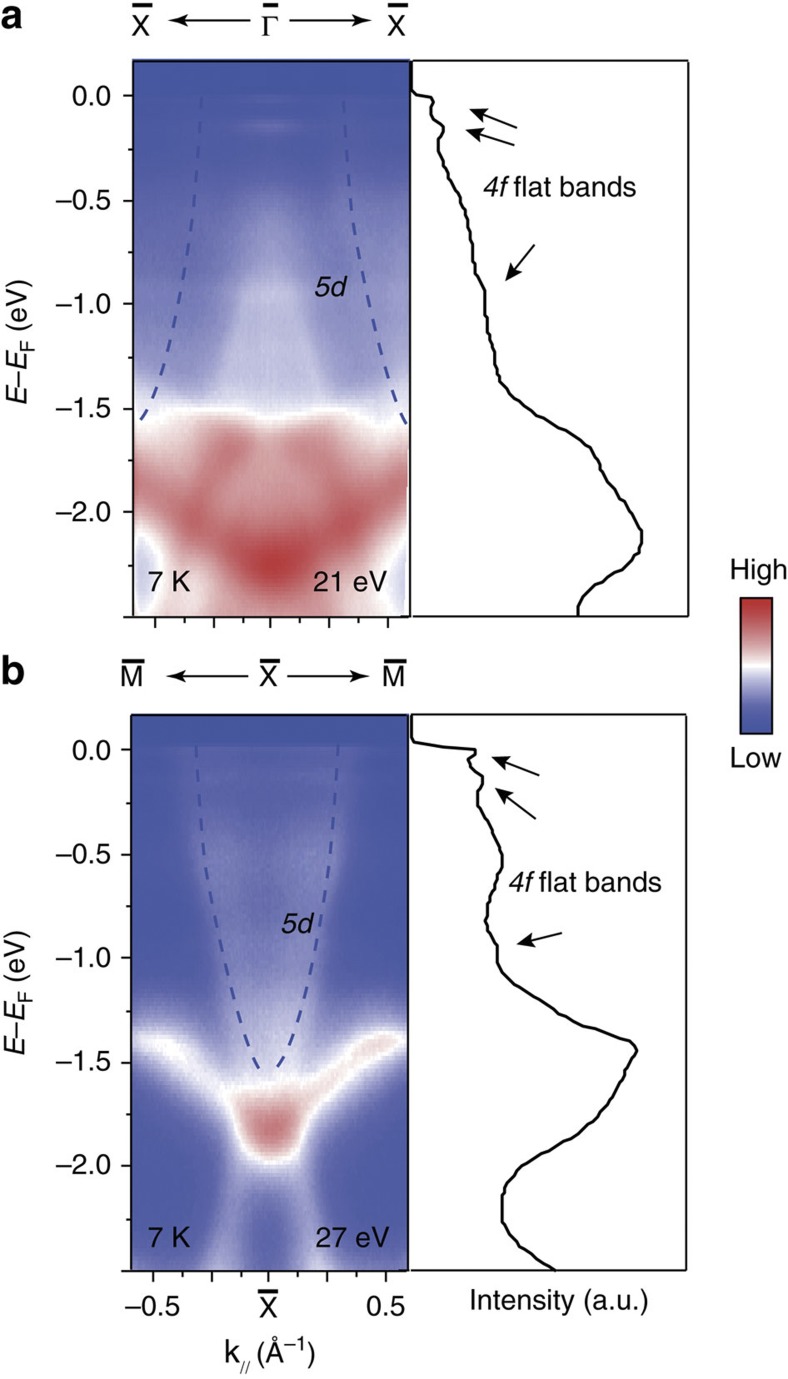
Valence band structure of SmB_6_. (**a**,**b**) Left panel: large energy scale photoemission intensity plot along the 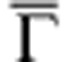
–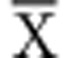 and 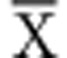
–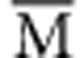 directions, respectively. Right panel: integrated EDCs to highlight the positions of the flat *4f* bands as marked by the arrows. Dashed lines in panels **a** and **b** indicate the Sm *5d* band that crosses *E*_F_ at high temperatures. Data were taken at 7 K with 21 and 27 eV photons, respectively, at Stanford Synchrotron Radiation Lightsource.

The photoemission intensities measured along 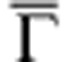
–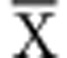 and 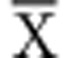
–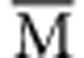 directions over a large energy scale are presented in Fig. 2a,b. Three flat bands can be observed at 18, 150 and 950 meV below the Fermi energy (*E*_F_), as marked in the integrated energy distribution curves (EDCs). They are the ^*6*^*H*_*5/2*_, ^*6*^*H*_*7/2*_ and ^*6*^*F* multiplets of the Sm^2+^ *4f*^*6*^→*4f*^*5*^ final state, respectively^10^,^22^. In addition, there is a highly dispersive band centred at 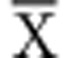 (dashed line). In previous ARPES studies^22^, it was ascribed to the bulk *5d* band based on the band calculations^23^.

### Dispersive in-gap states around 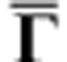 and 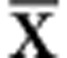


**Figure 3:**
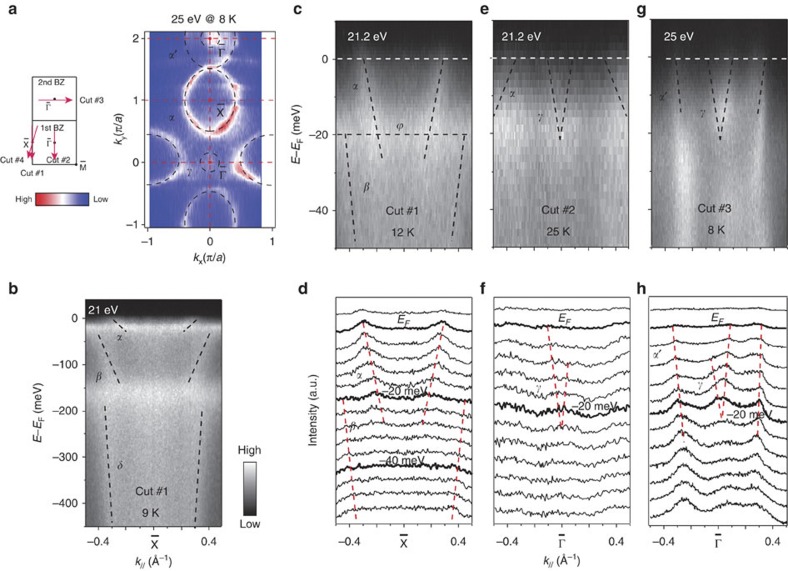
Dispersive in-gap states around 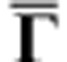 and 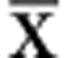. (**a**) Photoemission intensity map at the Fermi energy for SmB_6_ taken with 25 eV photons at Hiroshima Synchrotron Radiation Center. The intensity was integrated over a window of (*E*_F_−5 meV, *E*_F_+5 meV). The left panel is a sketch of the projected two-dimensional Brillouin zone. (**b**) Photoemission intensity plot over a large energy scale along cut #1 taken with 21 eV photons at Stanford synchrotron Radiation Lightsource. (**c**,**d**) The photoemission intensity plot and momentum distribution curves (MDCs) along cut #1 in the projected two-dimensional Brillouin zone as indicated in panel **a**, respectively. (**e**,**f**) The photoemission intensity plot and MDCs along cut #2, respectively. (**g**,**h**) The photoemission intensity plot and MDCs along cut #3, respectively. The dashed lines in panels **b**–**h** indicate the dispersions of various bands, ignoring band warping at crossings due to hybridization. The photon energies and temperatures for measurements are labelled in individual panels. The *α* and *γ* Fermi surfaces cover about 32.7 and 1.1% of projected two-dimensional Brillouin zone, respectively. The Fermi velocities of α and γ are ~0.24 and ~0.22 eV Å, respectively; the linear extrapolations of the *α* and *γ* band dispersions cross at 65±4 and 23±3 meV below *E*_F_, respectively.

To search for the in-gap states in SmB_6_, the photoemission intensity at *E*_F_ is collected over the surface projected Brillouin zone (Fig. 3a). Data were taken at 8 K, well into the KI phase. Indeed, four large oval-shaped Fermi surfaces are clearly observed around 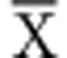, whose total area covers about 32.7% of the projected two-dimensional Brillouin zone, together with some spectral weight around 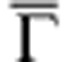. In the second Brillouin zone, the Fermi surface contour around 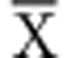 is weak while the spectral weight around 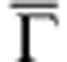 is much enhanced probably because of the matrix element effects of the photoemission process. The nature of these states can be further revealed in their dispersions. In Fig. 3b, we show the photoemission intensity plot over a larger energy scale along cut #1 across 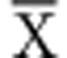. The aforementioned *5d*-like band is marked as *δ*, and the band between the two flat *4f* bands is referred to as *β*. More details of the near-*E*_F_ states and the hybridization gap are further illustrated in Fig. 3c. Here the hybridization gap is manifested by the suppressed spectral weight between *E*_F_ and the ^*6*^*H*_*5/2*_ *4f* band at −18 meV (referred to as *ϕ*). However, there is an in-gap dispersive band centred at 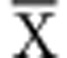 (referred to as *α*), which gives the large oval-shaped Fermi surface as shown in Fig. 3a. The dispersion of *α* is clearly visible in the momentum distribution curves (MDCs) in Fig. 3d as well, with Fermi crossings at ±0.29 Å^−1^. The band becomes obscure when it crosses *ϕ* and disappears below −30 meV. The photoemission intensity along a cut across 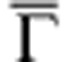 is shown in Fig. 3e, where the weak feature located at 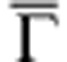 exhibits an electron-like band dispersion as further illustrated by their MDCs in Fig. 3f. This is more readily visible in the second Brillouin zone as shown in Fig. 3g,h. This band (referred to as *γ*) gives the tiny central electron pocket with a Fermi momentum of ~0.09 Å^−1^ in Fig. 3a. Its area is just about 1.1% of the projected two-dimensional Brillouin zone. The bright spot on band *ϕ* at 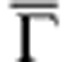 could be attributed to its crossing with *γ*, and the crossing of the two *γ* branches is estimated to occur at 23±3 meV. Furthermore, an oval-shaped Fermi surface *α*′ can be observed around 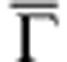 in the 2nd Brillouin zone, which shows almost the same size as the *α*-Fermi surface around 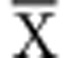, although it appears weaker in the 1st Brillouin zone. Its twofold symmetry and size indicate that it is most likely to be the shadow Fermi surface from the umklapp scattering of the *α*-Fermi surface around 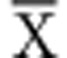 owing to the existence of a 1 × 2 surface reconstruction in this sample as reported in ref. 10 before. However, the superstructure spot of such a reconstruction is missing in the LEED pattern of our samples (Fig. 1d). Probably, it is too weak for the sensitivity of our LEED apparatus.

### *k*_*z*_ dependence of the electronic structure

**Figure 4:**
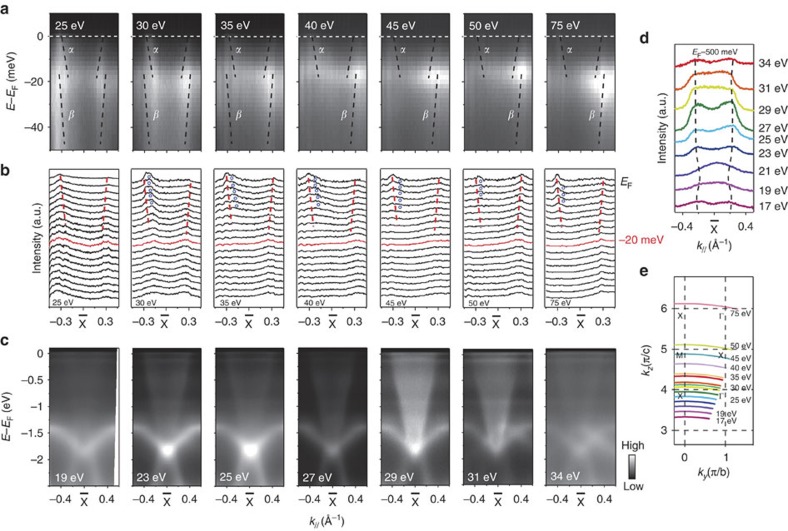
Photon energy dependence of the in-gap state around 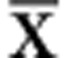. (**a**) Photon energy dependence of the photoemission intensity measured along cut #4 shown in Fig. 2a. The *α* and *β* band dispersions are indicated by the dashed lines. The bright regions correspond to the location where the 4*f* and 5*d* bands hybridize, which are particularly strong at some photon energies due to matrix element effects. Data were taken at 1 K at BESSY II. (**b**) The corresponding MDC plots of panel **a**. The red dashed lines are dispersions tracked from the MDC peak in the 25-eV data, and they are overlaid on data taken at other photon energies, indicating the lack of photon energy dependence. The blue circles refer to the shoulders in the MDCs that might be contributed by the bulk band. (**c**) The photoemission intensity measured along cut #1 in Fig. 2a for various photon energies. Data were taken at 7 K at Stanford Synchrotron Radiation Lightsource. (**d**) MDCs at −500 meV for data in panel **c**. (**e**) The sampled momentum cuts for various photon energies calculated with the inner potential 14 eV following ref. 10, indicating that the photon range that we used cover an extended Brillouin zone in *k*_*z*_.

Since the bulk SmB_6_ is an insulator based on the transport data and calculations, the in-gap states *α* and *γ* should be some metallic surface states. To further illustrate this, we varied photon energies to reveal the *k*_*z*_ dependence of the electronic structure. The photoemission intensity distribution across 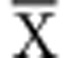 taken with various photon energies are presented in Fig. 4a. The photon energies we used cover a full *k*_*z*_ period in the extended Brillouin zone (Fig. 4e). The intensity distributions do not show any noticeable *k*_*z*_ dependence. In the corresponding MDC’s shown in Fig. 4b, we tracked the dispersion of the *α* band taken with 25 eV photons and overlaid it on the data taken with other photon energies, which clearly show that the dispersions and Fermi crossings of *α* are *k*_*z*_ independent. This behaviour indicates the surface origin of the *α* pockets. However, in addition, one could observe a shoulder feature that does show some photon energy dependence, which thus might be contributed by a bulk state. Furthermore, Fig. 4c shows the photoemission intensity distributions taken with various photon energies over a large energy scale across 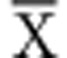. Since *k*_*x*_, *k*_*y*_ and *k*_*z*_ are equivalent in a cubic compound, the bulk *5d* bands are expected to have similar fast dispersions along *k*_*z*_ as those in *k*_*x*_ and *k*_*y*_ directions. However, at the first sight, the *5d*-like fast dispersive bands together with others do not show strong *k*_*z*_ dependence, for one can notice that the envelope of the photoemission intensity in Fig. 4c appears to be independent of the photon energies. Nevertheless, the bright interior of the intensity envelope for 29 and 31 eV data is a typical consequence of the projection of intensities from different *k*_*z*_ due to the poor *k*_*z*_ resolution of ARPES^24^. As is further illustrated by the broad MDCs taken at −500 meV around 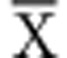 in Fig. 4d, the finite *k*_*z*_ resolution of ARPES spectral weight smears out the intensity distribution for the 5*d* band with a strong *k*_*z*_ dispersion. Combining these observations, we could conclude that the *α* band probably contains both surface and bulk contributions, while the *β* and *δ* bands are most probably originated from the bulk *5d* band.

### CD of the electronic structure

To study whether the surface states are topologically non-trivial or not, the chirality of their spin (**S**) and OAM shall be examined. This is another remarkable hallmark of the topological surface state: both the spin and OAM are interlocked and rotate with the electron momentum (**k**) and, their sum, the total angular momentum **J**, is a good quantum number. Spin-resolved ARPES requires long acquisition time because of its low count rate and, at this stage, it is not feasible for the weak surface state signal discussed above. Alternatively, instead of examining the chirality of the spin, one could examine the chirality of the OAM with the so-called CD of ARPES, namely, the difference of photoemission intensities taken with right circularly polarized (RCP) light and left circularly polarized (LCP) light. For Bi_2_Se_3_, it has been shown that photoemission CD signal is proportional to the inner product between OAM and light propagation vector^25^. This technique has been demonstrated to be a powerful tool to investigate the OAM texture of the surface states in TIs^25^,^26^.

**Figure 5:**
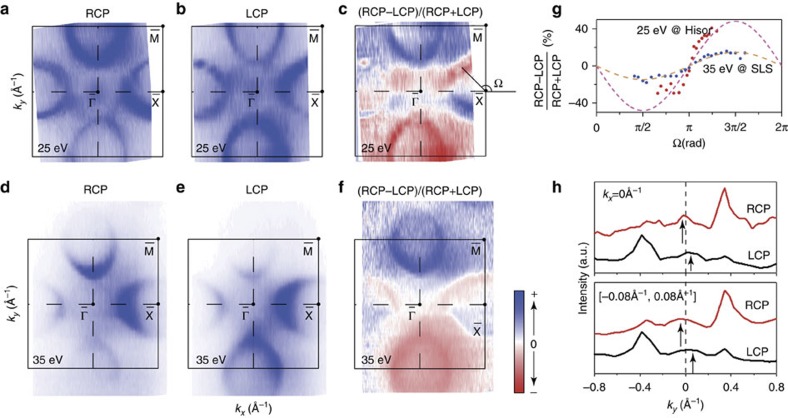
CD of the surface states at 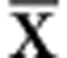 and 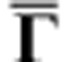. (**a**,**b**) Fermi surface maps of SmB_6_ taken with right circularly polarized (RCP) and left circularly polarized (LCP) light, respectively. The intensity was integrated over a window of (−5 meV, +5 meV). The data were taken at 8 K with 25 eV photons at Hiroshima Synchrotron Radiation Center (HiSOR). (**c**) The differential map of the RCP and LCP photoemission intensities in panels **a** and **b** that are normalized to their sum intensity. (**d**–**f**) The same as in panels **a**–**c**, but for another SmB_6−*x*_ sample with a slight boron deficiency. The intensity was integrated over a window of (−10 meV, +10 meV) and the data were taken at 27 K with 35 eV photons at Swiss Light Source. Note the *γ* band is not so visible here, probably because of its negligible matrix element at this photon energy. (**g**) The CD values taken along the right pocket in panel **c** (red dots) and in panel **f** (blue dots), where the polar angle Ω is defined within panel **c** with respect to 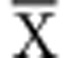. The dashed curves are sine function fits of the experimental data. (**h**) Top: the two MDCs along *k*_*y*_ taken at *E*_F_ and *k*_*x*_=0 with RCP and LCP light, respectively, where the CD of the *γ* band are indicated by the arrows. Bottom: analogous to the top except that the data are integrated over a *k*_*x*_ window of (−0.08 Å^−1^, 0.08 Å^−1^) to cover the *γ* pocket. The data were taken at 8 K with 25 eV photons at HiSOR.

Figures 5a and b show two Fermi surface maps taken with 25 eV RCP light and LCP light, respectively. Comparing the two data sets, we can clearly see the difference between the two intensity maps taken with different circular polarizations. The intensity of the upper electron pocket on the positive *k*_*y*_ side is higher than that in the lower electron pocket on the negative *k*_*y*_ side for the RCP data, while the LCP data exhibit an opposite behaviour. The electron pocket at the right side also shows the switching of high intensity regions between the positive *k*_*y*_ side and negative *k*_*y*_ side with different circularly polarized light. However, the electron pocket at the left side does not show strong CD, which is probably because of the asymmetric matrix element effects. Since the incident light is at 50° angle from *k*_*z*_ in the *k*_*x*_*–k*_*z*_ plane, the emission of the photoelectrons on the left and right Fermi surface sheets are asymmetric with respect to the light propagation direction. The CD can be more clearly represented by the normalized difference (RCP−LCP)/(RCP+LCP) in Fig. 5c. Figure 5d–f shows the similar results for another SmB_6−*x*_ sample measured with 35 eV photons. More specifically, Fig. 5g plots the CD values in Fig. 5c,f along the right *α-*Fermi pocket, which could be fitted by individual sinusoidal functions. The amplitude of CD is thus found to be photon energy dependent.

As for the *γ* band at 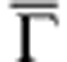, its weak intensity makes the CD effects not so obvious. However, for the data taken at 25 eV, Fig. 5h shows that there apparently exists certain intensity inversion between the RCP and LCP data in the MDCs across 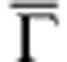 along the *k*_*y*_ axis. We can see that higher intensities appear on the negative *k*_*y*_ side for the RCP data but on the positive *k*_*y*_ side for the LCP data. Therefore, both *α* and *γ* bands show similar CD that is antisymmetric with respect to the *k*_*y*_=0 axis.

Many CD behaviours observed here resemble those of the non-trivial surface state found in Bi_2_Se_3_ before^25^,^26^, which indicates that both *α* and *γ* bands are likely to be topologically non-trivial. However, we notice that the CD behaviour of the *α* band is much more complicated than that of Bi_2_Se_3_. For example, the CD for the left *α* pocket is much weaker than the others, and the upper and lower *α* pockets exhibit different CD behaviours from that of the right *α* pocket. Particularly, the CD for both upper and lower *α* pockets does not change sign across the Brillouin zone boundary in Fig. 5c,f. In addition to certain final-state effect^27^, the complicated CD might in part be due to the coexistence of bulk and surface components in the *α* pocket. For example, if the bulk *5d* state and the surface state on the *α* pocket have different CD and their photoemission intensities vary differently with the emission angle and photon energy, one may expect asymmetric CD on left and right *α* pockets, and the photon energy-dependent CD amplitude shown in Fig. 5g. To fully understand these CD-ARPES data, such as the different functional forms of CD in the upper and right pockets, detailed density functional theory calculations of the OAM texture are required for future further studies, especially when there might be out-of-plane OAM component. Nevertheless, the pattern in our CD-ARPES data does suggest a non-trivial OAM texture (thus, also spin texture through spin-orbit coupling) for the *α* and *γ* bands, which is a necessary manifestation in photoemission if they are topologically non-trivial.

### Temperature dependence of the electronic structure

**Figure 6:**
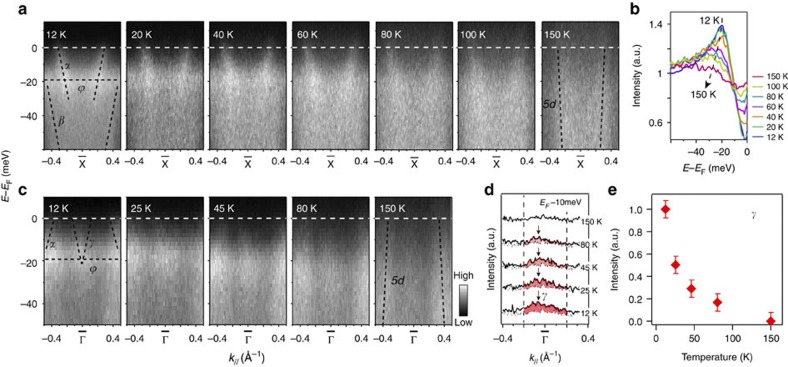
Temperature dependence of the photoemission data of the surface state around 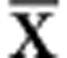. (**a**) Photoemission intensity of SmB_6_ along cut #1 in Fig. 2a at 12, 20, 40, 60, 80, 100 and 150 K. (**b**) Temperature dependence of the angle-integrated spectra of panel **a**, the Fermi cutoff effects were removed by dividing the angle-integrated spectra by the energy-resolution convoluted Fermi–Dirac distribution functions at individual temperatures. (**c**) Photoemission intensity of SmB_6_ along cut #2 in Fig. 2a at 12, 25, 45, 80 and 150 K. (**d**) Temperature dependence of the MDCs at 10 meV below *E*_F_ for data in panel **c**, and the shaded regions represent the difference from the 150-K data. (**e**) Area of the shaded region normalized by the 12-K area. The error bar is the relative uncertainty of the integrated photoelectron count. All data were taken with 21.2 eV photons.

If *α* and *γ* are topological surface states of a topological KI, they should vanish when the hybridization gap closes at high temperatures. Figure 6a presents temperature-dependent ARPES intensity measured across 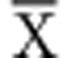. *α* stays almost unchanged at T<80 K, but its band velocity starts to increase above 80 K. Eventually, it appears that *α* and *β* merge into one highly dispersive band at 150 K, which fits the large-scale band dispersion shown in Fig. 2b. Therefore, *α*, *β* and *δ* could be the broken sections of the *5d* band induced by the *4f–5d* hybridization. However, one also sees that *α* goes straight through *ϕ* (Fig. 3c,d), which suggests that the *α*-Fermi surface probably contains contributions both from the not-fully gapped bulk *5d* band and from the topological surface state, although the latter disappears before ending at a Dirac point. This is also consistent with the conclusion that we draw from the photon energy dependence data.

The temperature evolution of the hybridization can be further demonstrated by the integrated EDCs of the images in Fig. 6a. After some processing described in the caption of Fig. 6, the resulting EDCs are presented in Fig. 6b. As temperature increases, the peak near −20 meV (derived from *ϕ*) is gradually suppressed, and the dip near *E*_F_ (a measure of the hybridization strength) is gradually filled. This indicates that the *4f* states become localized and completely lose coherence at T≈150 K. Consequently, they are decoupled with the *5d* bands and the hybridization disappears^28^,^29^. Similarly, in the photoemission intensity near 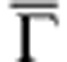 plotted in Fig. 6c, the *γ* band also vanishes with the hybridization gap at 150 K, which can be further visualized by the MDCs in Fig. 6d, and the integrated intensity in Fig. 6e as well. The thermal broadening effects from 12 to 80 K are not obvious in Fig. 6d; thus, the disappearance of the intensity at 150 K should be intrinsic. Therefore, the temperature dependencies of the surface states are consistent with the behaviours expected for a TKI.

## Discussion

Since the (001) surface Brillouin zone contains one 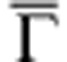 and two 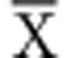 points on the zone boundary, we have two *α* bands enclosing two surface 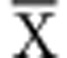 points but only one *γ* band around 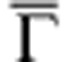. Regardless of whether or not *α* is a topologically trivial surface state, there will be totally an odd number of branches of surface states, which indicates that the whole system is topologically non-trivial. In fact, the basic electronic structure of these surface states qualitatively agrees with those topological surface states predicted in refs 16,17 remarkably well, although there are some complications in the CD that are still to be understood. In contrast, we note that unlike the case of weakly correlated systems, where theoretical calculations work quantitatively well for describing TIs^14^,^15^, there are some quantitative differences between our data and the calculations of SmB_6._ For example, the Fermi crossings of *α* and *γ* are different from the calculations, and the observed *α* Fermi surface has five to six times area of the calculated ones. The discrepancies might be partially because the chemical potentials used in theories are different from that in the real surface, and/or the actual cleaved surface is different from the ideal surface used in the calculations. Furthermore, these discrepancies also demonstrate that correlation might affect the topological surface states, since different calculations could vary somewhat depending on how the correlations were treated^16^,^17^. Finally, it is also intriguing to note that the Fermi momenta of the two surface states on the (101) surface are 0.0383 and 0.0955 Å^−1^, respectively as found in a recent quantum oscillation measurements of SmB_6_ (ref. 30), which are rather different from our measurements of the (001) surface (0.09 Å^−1^ for the small pocket near 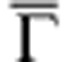 and 0.29 Å^−1^ along the short axis of the large oval Fermi surface).

In summary, we present a direct observation of the in-gap surface states in SmB_6_. The CD, and photon energy and temperature dependences of these in-gap states were systematically studied, which exhibit characteristic behaviours of a topological surface state for a KI, and thus support that SmB_6_ is a possible TKI. Our findings lay the foundation for understanding the anomalous transport properties of SmB_6_ and further exploration of the interplay between strong correlations and topological effects in TKI’s in general.

### Note added

After finishing this manuscript, we note that there are other three pieces of independent ARPES work on SmB_6_ (refs 31, 32, 33). Two of them report similar results^31^,^32^, except that our temperature dependence data agree with those reported in ref. 31, while ref. 32 suggests the surface states vanish at a much lower temperature of 30 K. The size of the *α* pocket measured here is similar to that reported in ref. 31 but much smaller than that reported in ref. 32. These discrepancies may be caused by different samples and requires further investigation. On the basis of data taken at 38 K, ref. 33 claims that the in-gap states around X must be bulk states. This partially agrees with our findings. However, the detailed data here taken at lower temperatures do indicate that the *α* band contains both surface and bulk components.

## Methods

### Sample synthesis

High-quality single crystals of SmB_6_ were grown either with the arc-melting technique or the Al-flux method. The arc melting technique was conducted by establishing a temperature gradient in the slow cooling process. First, the metal Sm were cut into small pieces and mixed with the boron powder in the ratio of Sm:B=1:6. The mixture was pressed into a pellet with the diameter of 1 cm and thickness of about 0.5 cm. Then the pellet was heated up in the water-cooled clean oven for arc melting. Welding was repeated for five times to ensure the sample uniformity. In the Al-flux method, a chunk of Sm (99.9%) together with the powders of Boron (99.99%) and Al (99.99%) were mixed with a ratio of 1:6:400. Then the mixture was loaded into an alumina crucible. The entire mixture was heated to 1,773 K and then maintained at 1,773 K for about 2 days before slowly cooling it down to 873 K at 5 K h^−1^. During all the heating progress, the mixture was kept in the circumstance with flowing Argon gas. The samples with Al flux were soaked in dense NaOH solution, and then washed with dilute HNO_3_ solution. Shiny crystals in the sizes of millimetre or sub-millimetre were obtained.

### ARPES measurement

The samples were cleaved along the (001) plane and measured under ultra-high vacuum of 3 × 10^−11^ torr. The in-house ARPES measurements were performed with SPECS UVLS discharge lamp (21.2 eV He-Iα light). The synchrotron ARPES experiments were performed at the Beamline 5-4 of Stanford Synchrotron Radiation Lightsource and the one-cubed ARPES station of BESSY II. The CD experiments were performed at Beamline 9 A of Hiroshima Synchrotron Radiation Center and the Surface and Interface Spectroscopy (SIS) beamline of Swiss Light Source. All data were taken with Scienta electron analysers, the overall energy resolution was better than 7 meV and the angular resolution was 0.3°.

## Author contributions

J.J., Z.R.Y., Z.S., S.Y.T., M.Xu, Q.Q.G., X.H.N., M.Xia and B.P.X. performed ARPES measurements. S.L. and Y.F.L. grew most of the samples and conducted sample characterization measurements, F. C. and X. H. C. grew the samples for the CD-ARPES measurement, J.J., T.Z. and D.L.F. analysed the ARPES data, T.Z., D.L.F., J.J., Z. S. and H.H.W wrote the paper. D.L.F. and H.H.W. are responsible for the infrastructure, project direction and planning.

## Additional information

**How to cite this article:** Jiang, J. *et al.* Observation of possible topological in-gap surface states in the Kondo insulator SmB_6_ by photoemission. *Nat. Commun.* 4:3010 doi: 10.1038/ncomms4010 (2013).

## References

[b1] Aeppli G. & Fisk Z. Kondo insulators. Comm. Condens. Matter Phys. 16, 155 (1992).

[b2] Riseborough P. Heavy fermion semiconductors. Adv. Phys. 49, 257–320 (2000).

[b3] Coleman P. Heavy Fermions: Electrons at the Edge of Magnetism, Handbook of Magnetism and Advanced Magnetic Materials Vol 1, 95–148Wiley (2007).

[b4] Menth A., Buehler E. & Geballe T. H. Magnetic and semiconducting properties of SmB_6_. Phys. Rev. Lett. 22, 295 (1969).

[b5] Allen J. W., Batlogg B. & Wachter P. Large low-temperature Hall effect and resistivity in mixed-valent SmB_6_. Phys. Rev. B 20, 4807–4813 (1979).

[b6] Cooley J. C., Aronson M. C., Fisk Z. & Canfield P. C. SmB_6_: Kondo insulator or exotic metal? Phys. Rev. Lett. 74, 1629 (1995). 10059077 10.1103/PhysRevLett.74.1629

[b7] Nanba T. *et al.* Gap state of SmB_6_. Physica B 186–188, 440–443 (1993).

[b8] Nyhus P., Cooper S. L., Fisk Z. & Sarrao J. Low-energy excitations of the correlation-gap insulator SmB_6_: a light-scattering study. Phys. Rev. B 55, 12488 (1997).

[b9] Alekseev P. A. *et al.* Magnetic excitations in SmB_6_ single crystals. Physica B 186–188, 384–386 (1993).

[b10] Miyazaki H. *et al.* Momentum-dependent hybridization gap and dispersive in-gap state of the Kondo semiconductor SmB_6_. Phys. Rev. B 86, 075105 (2012).

[b11] Curnoe S. & Kikoin K. A. Electron self-trapping in intermediate-valent SmB_6_. Phys. Rev. B 61, 15714 (2000).

[b12] Dzero M. *et al.* Topological Kondo insulators. Phys. Rev. Lett. 104, 106408 (2010). 20366446 10.1103/PhysRevLett.104.106408

[b13] Dzero M. *et al.* Theory of topological Kondo insulators. Phys. Rev. B 85, 045130 (2012).

[b14] Hasan M. Z. & Kane C. L. Colloquium: topological insulators. Rev. Mod. Phys. 82, 3045 (2010).

[b15] Qi X. L. & Zhang S. C. Topological insulators and superconductors. Rev. Mod. Phys. 83, 1057 (2011).

[b16] Takimoto T. SmB_6_: a promising candidate for a topological insulator. J. Phys. Soc. Jpn 80, 123710 (2011).

[b17] Lu F. *et al.* Correlated topological insulators with mixed valence. Phys. Rev. Lett. 110, 096401 (2013). 23496729 10.1103/PhysRevLett.110.096401

[b18] Kim D. J., Grant T. & Fisk Z. Limit cycle and anomalous capacitance in the Kondo insulator SmB_6_. Phys. Rev. Lett. 109, 096601 (2012). 23002866 10.1103/PhysRevLett.109.096601

[b19] Wolgast S. *et al.* Low temperature surface conduction in the Kondo insulator SmB_6_. Preprint at http://arxiv.org/abs/1211.5104 (2012).

[b20] Zhang X. *et al.* Hybridization, inter-ion correlation, and surface states in the Kondo insulator SmB_6_. Phys. Rev. X 3, 011011 (2013).

[b21] Botimer J. *et al.* Robust surface Hall effect and nonlocal transport in SmB_6_: indication for an ideal topological insulator. Preprint at http://arxiv.org/abs/ 1211.6769 (2012).

[b22] Denlinger J. D. *et al.* Advances in photoemission spectroscopy of f-electron materials. Physica B 281&282, 716–722 (2000).

[b23] Massidda S. *et al.* Electronic structure of divalent hexaborides. Z. Phys. B 102, 83–89 (1997).

[b24] Ye Z. R. *et al.* Orbital selective correlations between nesting/scattering/Lifshitz transition and the superconductivity in AFe_1-x_Co_x_As (A=Li, Na). Preprint at http://arxiv.org/abs/1303.0682 (2013).

[b25] Park S. R. *et al.* Chiral orbital-angular momentum in the surface state of Bi_2_Se_3_. Phys. Rev. Lett. 108, 046805 (2012). 22400876 10.1103/PhysRevLett.108.046805

[b26] Wang Y. H. *et al.* Observation of a warped helical spin texture in Bi_2_Se_3_ from circular dichroism angle-resolved photoemission spectroscopy. Phys. Rev. Lett. 107, 207602 (2011). 22181776 10.1103/PhysRevLett.107.207602

[b27] Scholz M. R. *et al.* Reversal of the circular dichroism in angle-resolved photoemission from Bi_2_Te_3_. Phys. Rev. Lett. 110, 216801 (2013). 23745908 10.1103/PhysRevLett.110.216801

[b28] Nozawa S. *et al.* Ultrahigh-resolution and angle-resolved photoemission study of SmB_6_. J. Phys. Chem. Solid 63, 1223–1226 (2002).

[b29] Souma S. *et al.* Direct observation of pseudogap of SmB_6_ using ultrahigh—resolution photoemission spectroscopy. Physica B 312-313, 329–330 (2002).

[b30] Li G. *et al.* Quantum oscillations in Kondo Insulator SmB_6_. Preprint at http://arxiv.org/abs/1306.5221 (2013).

[b31] Xu N. *et al.* Surface and bulk electronic structure of the strongly correlated system SmB_6_ and implications for a topological Kondo insulator. Phys. Rev. B 88, 121102(R) (2013).

[b32] Neupane M. *et al.* Surface electronic structure of the topological Kondo Insulator candidate correlated electron system SmB_6_. Nat. Commun 4, 2991 (2013). 24346502 10.1038/ncomms3991

[b33] Frantzeskakis E. *et al.* Kondo hybridization and the origin of metallic states at the (001) surface of SmB_6_. Preprint at http://arxiv.org/abs/1308.0151 (2013).

